# Role of Minor Ta Substitution on Thermal Behavior and Soft Magnetic Properties of Co-Fe-Mo-Si-B Metallic Glass Ribbon

**DOI:** 10.3390/ma18081828

**Published:** 2025-04-16

**Authors:** Peipei Shen, Yanan Gao, Shuyan Zhang, Hua Chen, Pengfei Wang, Yangzhi Xue, Hongbo Zhou, Danyue Ma, Jixi Lu

**Affiliations:** 1 Hangzhou Institute of Extremely-Weak Magnetic Field Major National Science and Technology Infrastructure, Hangzhou 310052, Chinazsy19930524@sina.com (S.Z.); chhc0624@163.com (H.C.); pengfeibg@gmail.com (P.W.); madanyue0419@buaa.edu.cn (D.M.); lujixi@buaa.edu.cn (J.L.); 2 Key Laboratory of Ultra-Weak Magnetic Field Measurement Technology, Ministry of Education, School of Instrumentation and Optoelectronic Engineering, Beihang University, Beijing 100191, China; xyz_buaa@buaa.edu.cn; 3 State Key Laboratory of Nonlinear Mechanics, Institute of Mechanics, Chinese Academy of Sciences, Beijing 100190, China; zhouhb@imech.ac.cn

**Keywords:** cobalt-based metallic glasses, thermal behavior, glass-forming ability, magnetic performance

## Abstract

Cobalt-based metallic glasses have sparked intensive attention because of their extraordinary properties. In this work, a series of Co_66_Fe_4_Mo_2-*x*_Ta*_x_*Si_16_B_12_ (*x* = 0, 0.5, 1.0, 1.5, 2.0) metallic glass ribbons were systematically designed to investigate the influence of the minor Ta substitution for Mo on the thermal behavior and magnetic performance. The results reveal that the width of the supercooled liquid region initially increases with Ta content, reaching 98 K at *x* = 1.0, and subsequently decreases with further Ta addition. It indicates that the Co_66_Fe_4_Mo_1.__0_Ta_1.__0_Si_16_B_12_ alloy has the optimal glass-forming ability. Moreover, the crystallization onset temperature and crystallization peak temperature of all as-quenched ribbons were improved with the Ta content *x* increasing to 2.0, which is due to the higher melting temperature of the element Ta (3290 K). In addition, these ribbons exhibit outstanding soft magnetic properties, including ultralow coercivity (*H*_c_ < 1.1 A/m) and moderate saturation magnetization, which indicates that these ribbons are suitable for magnetic shielding. These results offer valuable insights into the design of soft magnetic metallic glass.

## 1. Introduction

Since Au-Si metallic glass (MG) was first reported in 1960, extensive research efforts have led to the development of a large number of metallic glasses (MGs) with a long-range disordered atomic arrangement structure [1]. These amorphous alloys exhibit extraordinary properties arising from their inherently non-periodic structures [2,3]. Cobalt-based metallic glasses (Co-based MGs), available in the forms of ribbons, bulk materials, and microfibers, represent an important category of advanced materials. They combine exceptional soft magnetic properties [4], excellent thermodynamic stability [5,6], and remarkable ultra-high strength [7,8]. Cobalt-based MGs have emerged as superior alternatives to iron-based amorphous/nanocrystalline alloys, primarily due to their inherently lower coercivity and power loss. These advantageous properties originate from their near-zero magnetostriction and magneto-crystalline anisotropy [9,10]. For example, Liang et al. reported that Co–Y–B MGs exhibit lower coercivity (*H*_c_) than Fe–Y–B MGs [11,12]. The excellent soft magnetic properties and low power-loss characteristics of Co-based MGs make them ideal candidates for applications such as magnetic shielding, magnetic sensors, and power devices [13]. For example, polystyrene-grafted Co-based MG composites with high permeability and low power loss are developed for magnetic shielding, which achieves a significant suppression of magnetic noise [14]. In addition, the Co-Fe-Mo-Si-B MG microfibers were successfully fabricated by the modified melt-spinning technique for flexible electromagnetic shielding [15].

In recent years, various Co-based MG systems have been successfully synthesized, such as Co-Fe-Si-B, Co-Er-B, and Co-Fe-B-P-C [16,17,18]. The optimization of glass-forming ability (GFA) in these alloys has been achieved through compositional design, based on fundamental principles such as Inoue’s three empirical rules and advanced microalloying techniques [19,20]. It has been demonstrated that the proper selection of the constituent elements significantly affects the GFA and plays a critical role in determining the soft magnetic properties of MGs [21]. Consequently, one effective approach to enhance the GFA, and soft magnetic performance of Co-based MGs is through microalloying or the minor substitution of specific elements to optimize the chemical composition [22,23]. For example, previous studies have found that optimizing the ratio of elements B and Si can tune the thermal behavior and improve the GFA of Co-based MGs [24,25]. In addition, Neamtu et al. improved the crystallization temperature of Co-Fe-Ni-Si-B MG powders by substitution of Si or B with Zr or Ti [26].

The addition of tantalum (Ta), a high-melting-point transition metal, often tailors the thermal behavior of Co-based MGs while maintaining their excellent soft magnetic properties. For example, the Co_43_Fe_20_Ta_5.__5_B_31.__5_ bulk MG exhibits a glass transition temperature as high as 910 K and an exceptional coercivity of only 0.25 A/m [27]. Furthermore, Nickjeh et al. reported that microalloying with Ta dramatically enhances the GFA of mechanically alloyed Co-based MGs [28]. The Co_66_Fe_4_Mo_2_Si_16_B_12_ MG has excellent soft magnetic properties and moderate saturation magnetization, which is an ideal candidate for magnetic shielding and magnetic sensors [29]. However, the literature lacks investigations connecting the minor addition of the Ta with the thermal behavior and soft magnetic properties of the Co_66_Fe_4_Mo_2_Si_16_B_12_ MG.

In the present study, we systematically designed and fabricated new Co_66_Fe_4_Mo_2-*x*_Ta*_x_*Si_16_B_12_ (*x* = 0, 0.5, 1.0, 1.5, 2.0) MGs. The effects of minor Ta replacing Mo on the thermal behaviors and magnetic performance were investigated. The thermal behaviors of Co_66_Fe_4_Mo_2-*x*_Ta*_x_*Si_16_B_12_ MGs, including the glass transition temperature (*T*_g_), crystallization onset temperature (*T*_x_), and crystallization peak temperature (*T*_p_), are improved with the increase in Ta content *x*. Especially, the Co_66_Fe_4_Mo_1.__0_Ta_1.__0_Si_16_B_12_ has the best GFA in this alloy system. Furthermore, all of the as-quenched Co_66_Fe_4_Mo_2-*x*_Ta*_x_*Si_16_B_12_ MGs ribbons exhibit outstanding soft magnetic properties, characterized by an exceptionally low coercivity (*H*_c_) of less than 1.1 A/m.

## 2. Materials and Methods

**Figure 1:**
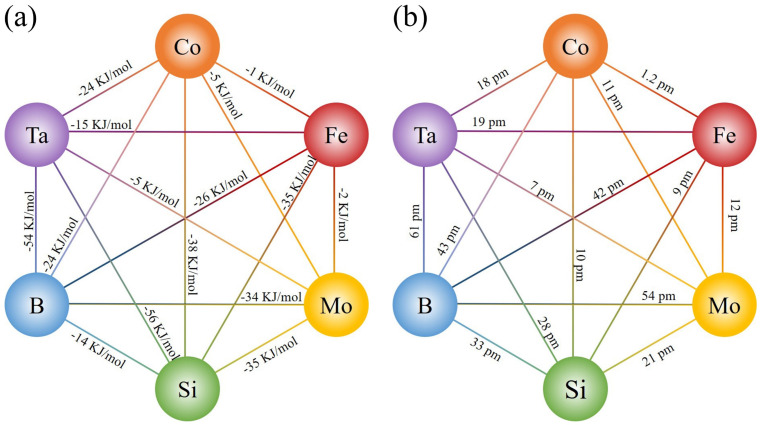
(**a**) Mixing enthalpy and (**b**) atomic radius differences among alloy components.

As illustrated in Figure 1a,b, the negative mixing enthalpy and the difference in radius of Ta with other elements is higher than that of Mo in the Co-Fe-Mo-Si-B system. According to Inoue’s criteria, the minor substitution of Ta for Mo is expected to improve the GFA of the alloy system [30,31]. In addition, the minor substitution of the Ta can also influence the thermal behavior of Co-Fe-Mo-Si-B MG. Therefore, Co_66_Fe_4_Mo_2-*x*_Ta*_x_*Si_16_B_12_ MGs with different amounts of Ta content were designed and fabricated in this study.

The homogeneous alloy ingots on the base of Co_66_Fe_4_Mo_2-*x*_Ta*_x_*Si_16_B_12_ (*x* = 0, 0.5, 1.0, 1.5, 2.0) were produced by arc melting a mixture of pure metals and pure metalloids (purity ≥ 99.5 wt.%) multiple times. The densities of the alloy ingots with different Ta content measured by the Archimedes method are 7.78, 7.89, 7.92, 7.99, and 8.06 g/cm^3^, which were used to calculate the magnetic flux density (*B*) of alloy ribbons. Alloy ribbons were prepared by the single-roller melt-spinning process, which has a cooling rate of 10^6^ K/s, under an argon atmosphere to ensure rapid solidification and suppress crystallization [32]. The amorphous structure of alloy ribbons was identified by X-ray diffraction (XRD) using Bruker D8 Advance (Billerica, MA, USA) with a scanning speed of 1 deg/min in theta–2theta scan mode. Thermal properties, including *T*_g_, *T*_x_, and *T*_p_, were determined by differential scanning calorimetry (DSC) using a TGA/DSC 3+ (METTLER, Greifensee, Switzerland) in the temperature range from 323 K to 1273 K under a constant flow of high-purity nitrogen gas [33]. To obtain the saturation magnetic flux density (*B*_s_), hysteresis loop tests were performed on the alloy ribbons at room temperature by MPMS 3 (Quantum Design, San Diego, CA, USA). In addition, the coercivity (*H*_c_) of the ribbons was determined under an applied DC magnetic field of 80 A/m using the RIKEN BHS-40 DC *B*-*H* loop tracer (Tokyo, Japan) [34].

## 3. Results and Discussions

**Figure 2:**
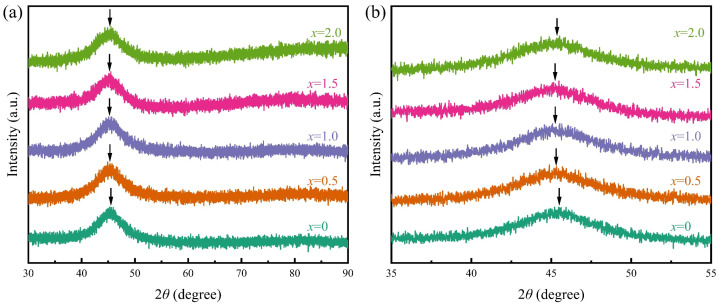
(**a**) XRD patterns of as-quenched Co_66_Fe_4_Mo_2-*x*_Ta*_x_*Si_16_B_12_ (*x* = 0, 0.5, 1.0, 1.5, 2.0) alloy ribbons. (**b**) Enlarged XRD patterns in the 2*θ* angular range of 35–55°.

As shown in Figure 2a, the XRD patterns of the as-quenched Co_66_Fe_4_Mo_2-*x*_Ta*_x_*Si_16_B_12_ (*x* = 0, 0.5, 1.0, 1.5, 2.0) alloy ribbons reveal a typical diffused diffraction peak characteristic of an amorphous structure without any Bragg reflection peak corresponding to crystalline phases, which confirms that all ribbons prepared in this study maintain a long-range disordered atomic arrangement and have the fully glassy structure. Figure 2b is the enlarged XRD patterns of Figure 2a in the 2θ angular range of 35–55°. Detailed analysis of the position of the diffused peak, which was marked by the arrow in Figure 2b, reveals a non-monotonic dependence with increasing Ta content. Specifically, the position of the diffused peak shifts toward the lower 2theta angle side for *x* = 0.5 and 1.0. However, the position of the diffused peak shifts back toward a higher 2theta angle for higher Ta content *x*. In particular, the lowest position of the diffused peak observed at Ta content *x* = 1.0 implies the maximum atomic distance appears. This indicates that the atomic distance can be effectively tailored by Ta addition, which is similar to the Fe-Co-based MG [35,36].

**Figure 3:**
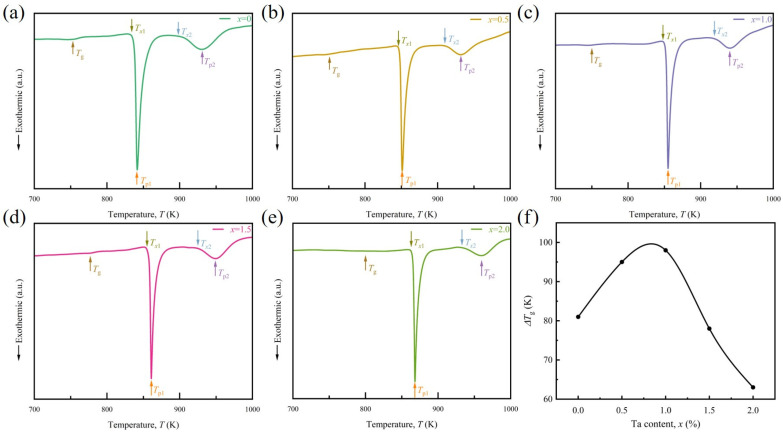
DSC curves of as-quenched Co_66_Fe_4_Mo_2-*x*_Ta*_x_*Si_16_B_12_ metallic glass ribbons. (**a**) *x* = 0. (**b**) *x* = 0.5. (**c**) *x* = 1.0. (**d**) *x* = 1.5. (**e**) *x* = 2.0. (**f**) Changes in the supercooled liquid region Δ*T*_x_ with the Ta content *x*.

To investigate the thermal behaviors of as-quenched Co_66_Fe_4_Mo_2-*x*_Ta*_x_*Si_16_B_12_ (*x* = 0, 0.5, 1.0, 1.5, 2.0) MG ribbons, DSC measurements were performed at a heating rate of 20 K/min. Figure 3a–e shows the DSC results of Co_66_Fe_4_Mo_2-*x*_Ta*_x_*Si_16_B_12_ (*x* = 0, 0.5, 1.0, 1.5, 2.0) MG ribbons. There is a glass transition before the crystallization of the fabricated MG ribbons, which can be seen from the endothermic reaction. It should be noted that the *T*_g_ of the Co_66_Fe_4_Mo_2-*x*_Ta*_x_*Si_16_B_12_ (*x* = 0, 0.5, 1.0) MG ribbons show almost no clearly change, remaining around 750 K (for details, see Table 1), which is in agreement with previous work [37]. However, the *T*_g_ increases significantly when the Ta content *x* is 1.5 or 2.

In addition, all ribbons have undergone two crystallization processes, which are reflected by two separated exothermic peaks of the DSC curves. The corresponding thermal parameters of ribbons are listed in Table 1. For each ribbon, the exothermic heat of the first crystallization peak is higher than the second one, implying that the first stage dominates the crystallization process of MG ribbons. Moreover, the crystallization behaviors have an evident relationship with Ta content *x* in this alloy system (seen in Figure 3 and Table 1). For example, the primary crystallization onset temperature *T*_x1_ and primary crystallization peak temperature *T*_p1_ show the tendency to increase with Ta content *x*, attributing to the higher melting temperature of element Ta (3290 K) compared to the element Mo (2896 K).

Notably, the width of supercooled liquid region Δ*T*_x_, defined as the range between the *T*_g_ and the *T*_x1_, increases from 81 K with *x* = 0, to 98 K for *x* = 1.0 in our experiment. With further increasing Ta content from *x* = 1.0 to 2.0, the width of Δ*T*_x_ decreases (shown in Figure 3e). Hence, Co_66_Fe_4_Mo_1.__0_Ta_1.__0_Si_16_B_12_ exhibits the best GFA in this alloy system.

**Figure 4:**
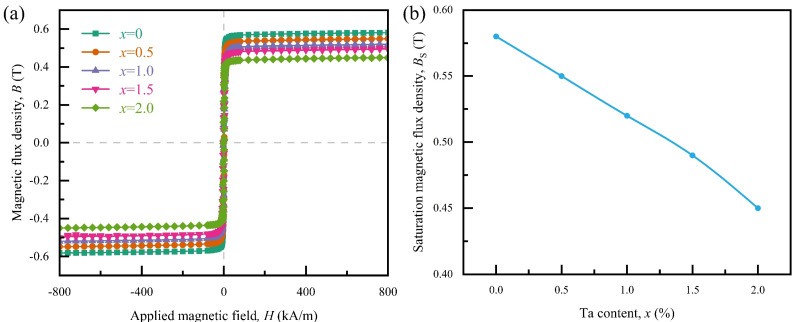
(**a**) Hysteresis loops of Co_66_Fe_4_Mo_2-*x*_Ta*_x_*Si_16_B_12_ metallic glass ribbons measured at 300 K. (**b**) The Ta content *x* dependence of the saturation magnetic flux density *B*_s_.

Figure 4a presents the hysteresis loops of Co_66_Fe_4_Mo_2-*x*_Ta*_x_*Si_16_B_12_ MG ribbons in an external magnetic field up to 800 kA/m at 300 K, which shows the typical soft-magnetic property. The Co_66_Fe_4_Mo_2_Si_16_B_12_ MG ribbon shows the highest saturation magnetic flux density *B*_s_ value of 0.58 T, which is consistent with the previous studies [38,39,40,41]. The *B*_s_ of Co_66_Fe_4_Mo_2-*x*_Ta*_x_*Si_16_B_12_ MG ribbons slightly decrease from about 0.58 T to 0.45 T as Ta content *x* increases from 0 to 2, which indicates that the addition of element Ta will lead to a decrease in *B*_s_. In addition, the dependence of *B*_s_ on Ta content *x* for the alloy system is shown in Figure 4b. It can be observed that the *B*_s_ decreases approximately linearly with the increasing Ta content *x*. For the Co-Fe MG system, the *B*_s_ is mainly influenced by the average atomic magnetic moment of Fe [42,43]. Due to the higher mixing enthalpy of Ta with Fe compared to Mo (shown in Figure 1a), the average atomic magnetic moments of Fe may decrease uniformly with increasing Ta content *x*, resulting in a linear decrease in the *B*_s_ of the alloy system with increasing Ta content *x*.

**Figure 5:**
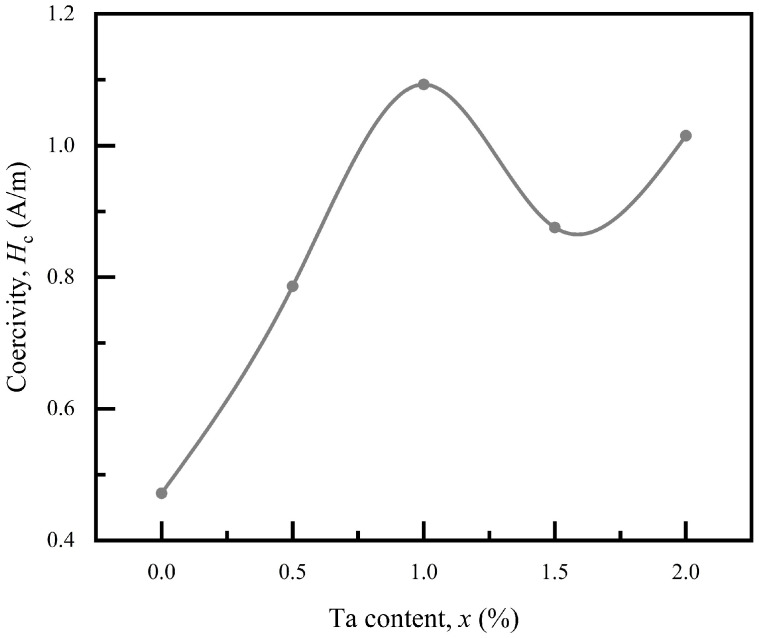
The coercivity *H*_c_ as a function of Ta content *x* at room temperature.

Coercivity is an important parameter of soft magnetic materials. The relationship between coercivity and Ta content *x* for Co_66_Fe_4_Mo_2-*x*_Ta*_x_*Si_16_B_12_ MG ribbons is presented in Figure 5. Compared with the Ta-free MG ribbon, the *H*_c_ of the addition of Ta with *x* = 0.5–2.0 deteriorated slightly but remained below 1.1 A/m, indicating these ribbons have excellent soft magnetic properties. In addition, it should be noted that the *H*_c_ is not monotonically changing with the increase in Ta content *x*, which implies that the addition of Ta has a complex effect on *H*_c_ in this alloy system.

## 4. Conclusions

In this work, the Co_66_Fe_4_Mo_2-*x*_Ta*_x_*Si_16_B_12_ (*x* = 0, 0.5, 1.0, 1.5, 2.0) MG ribbons are designed and fabricated. The roles of the minor substitution of Ta for Mo on the thermal behaviors and magnetic performance were investigated in detail. The as-quenched ribbons have high *T*_x1_ in the range of 834–864 K, which monotonically increases with the increase in Ta content *x*. Moreover, the Co-Fe-Mo-Ta-Si-B MG ribbons have the best GFA in this alloy system with the supercooled liquid region Δ*T*_x_ = 98 K when Ta content *x* = 1.0. In addition, all alloy ribbons exhibit outstanding soft magnetic properties at room temperature, characterized by low coercivity (*H*_c_ < 1.1 A/m) and moderate saturation magnetization. Despite a slight decrease in *B*_s_ with increasing Ta content, the overall soft magnetic performance remains promising for practical applications, such as magnetic shielding.

## Figures and Tables

**Table 1:** Thermal parameters of Co_66_Fe_4_Mo_2-x_Ta_x_Si_16_B_12_ metallic glass ribbons.

Ta Content *x*	*T*_g_ (K)	*T*_x1_ (K)	*T*_p1_ (K)	*T*_x2_ (K)	*T*_p2_ (K)	Δ*T*_x_ (K)
0	753	834	841	898	931	81
0.5	751	846	851	910	932	95
1.0	750	848	855	919	940	98
1.5	777	855	860	925	949	78
2.0	800	864	868	933	960	63

## Data Availability

The original contributions presented in this study are included in the article. Further inquiries can be directed to the corresponding author.
